# A deep learning framework for identifying and segmenting three vessels in fetal heart ultrasound images

**DOI:** 10.1186/s12938-024-01230-2

**Published:** 2024-04-02

**Authors:** Laifa Yan, Shan Ling, Rongsong Mao, Haoran Xi, Fei Wang

**Affiliations:** 1 College of Information Engineering, Zhejiang University of Technology, Hangzhou, Zhejiang China; 2 Hangzhou Institute of Medicine, Chinese Academy of Sciences, Hangzhou, Zhejiang China; 3 The Center of Four-Dimensional Ultrasound, Affiliated Xiaoshan Hospital, Hangzhou Normal University, Hangzhou, Zhejiang China

**Keywords:** Medical image segmentation, Congenital heart disease, Ultrasound image analysis, Deep learning, Prenatal heart examination

## Abstract

**Background:**

Congenital heart disease (CHD) is one of the most common birth defects in the world. It is the leading cause of infant mortality, necessitating an early diagnosis for timely intervention. Prenatal screening using ultrasound is the primary method for CHD detection. However, its effectiveness is heavily reliant on the expertise of physicians, leading to subjective interpretations and potential underdiagnosis. Therefore, a method for automatic analysis of fetal cardiac ultrasound images is highly desired to assist an objective and effective CHD diagnosis.

**Method:**

In this study, we propose a deep learning-based framework for the identification and segmentation of the three vessels—the pulmonary artery, aorta, and superior vena cava—in the ultrasound three vessel view (3VV) of the fetal heart. In the first stage of the framework, the object detection model Yolov5 is employed to identify the three vessels and localize the Region of Interest (ROI) within the original full-sized ultrasound images. Subsequently, a modified Deeplabv3 equipped with our novel AMFF (Attentional Multi-scale Feature Fusion) module is applied in the second stage to segment the three vessels within the cropped ROI images.

**Results:**

We evaluated our method with a dataset consisting of 511 fetal heart 3VV images. Compared to existing models, our framework exhibits superior performance in the segmentation of all the three vessels, demonstrating the Dice coefficients of 85.55%, 89.12%, and 77.54% for PA, Ao and SVC respectively.

**Conclusions:**

Our experimental results show that our proposed framework can automatically and accurately detect and segment the three vessels in fetal heart 3VV images. This method has the potential to assist sonographers in enhancing the precision of vessel assessment during fetal heart examinations.

## Introduction

Congenital heart disease (CHD) is the most prevalent birth defects, accounting for approximately 28% of all congenital abnormalities [1–3]. Research has reported that the incidence of CHD is around 1% in the global birth population [1–3]. CHD encompasses a spectrum of anatomical anomalies that result from disruptions or developmental irregularities in the formation of the fetal heart and major blood vessels during embryonic development [4]. Notably, it is the leading cause of neonatal mortality [4, 5]. Disturbances in fetal heart and major blood vessels can occur during the early stages of pregnancy, typically within the first 2–3 months, and have the potential to impair the normal growth and function of the infant's heart. Therefore, the early diagnosis of CHD is imperative and holds paramount significance in providing essential medical intervention and mitigating health risks for infants affected by this condition.

Ultrasound has emerged as the primary imaging modality for fetal heart examination thanks to its cost-effectiveness, lack of radiation exposure, and minimal side effects [6, 7]. During an ultrasound examination of the fetal heart, multiple cardiac planes should be carefully examined to thoroughly assess the cardiac four-chamber and vessels [8, 9]. The three-vessel view (3VV) is a critical cardiac plane that reveals the structure and function of the three major vessels of the fetal heart—the pulmonary artery, aorta, and superior vena cava [10]. Some typical cardiac anomalies that may appear normal in the four-chamber view are frequently identified in the 3VV, such as complete transposition of the great arteries, Tetralogy of Fallot, and pulmonary atresia with ventricular septal defect [11]. Therefore, a precise evaluation of the 3VV can improve the detection rate of significant cardiac malformations. However, the effectiveness of ultrasound diagnoses heavily relies on the experience and expertise of physicians, often leading to subjective ultrasound interpretations [6]. In cases where physicians lack sufficient experience, there is a risk of underdiagnosis or misdiagnosis. Experienced sonographers may also face challenges in making accurate diagnoses when dealing with complex examination procedures and a large volume of patients. Therefore, the development of an automated and reliable diagnostic tool capable of assessing cardiac vascular structures during fetal heart examinations is highly desired. Such a tool could significantly alleviate the workload of physicians and assist clinical physicians in performing more precise and efficient early CHD screening.

In recent years, deep learning has made remarkable progress in the field of medical image segmentation because of its powerful ability to autonomously learn image features and perform pixel-level classification [12–14]. One notable architecture is the Fully Convolutional Network (FCN), which consists of multiple convolutional layers and fully connected layers [15]. FCN leverages the deconvolution technique to restore the final feature map to the dimension of the input image, enabling pixel-level predictions and effectively addressing the challenge of semantic image segmentation. U-Net is another network that has been widely adopted for various segmentation tasks [16]. It is named from its U-shaped architecture characterized by an end-to-end encoder–decoder structure. The encoder gradually reduces the spatial dimension of the input image while extracting features. The decoder is responsible for upsampling the feature maps and progressively increasing the spatial dimension with the help of transposed convolutional layers. Recently, researchers have introduced several innovative techniques to enhance feature extraction and decoding capabilities, including the integration of multiple model architectures, the incorporation of residual pathways, and the utilization of attention mechanisms, etc. [17–19]. For example, Zhou et al. [20] proposed U-Net++, which integrates features of varying scales through a cascade of densely interconnected skip connections. This method minimizes semantic loss between feature maps and labels, enhancing the network's ability to capture salient information effectively. Additionally, Oktay et al. [21] proposed Attention U-Net, which introduces attention gates to suppress irrelevant regions and enhance valuable salient features crucial to the target. To address the challenge of multi-scale image segmentation Chen et al. [22] proposed Deeplabv3, which introduces dilated convolutions and ASPP (Atrous Spatial Pyramid Pooling) techniques to maintain the feature map size while effectively controlling the receptive field.

Deep learning has been applied in the field of fetal echocardiography for various tasks, including standard plane identification from a sequence of fetal heart images [23–25], detection of abnormal structures [26–29], and segmentation of cardiac structures [30–33]. Most studies designed for fetal cardiac structure segmentation have focused on the four-chamber view of the fetal heart [30, 31, 33]. In this study, we aim to develop a deep-learning based framework for the accurate segmentation of the three vessels within the three-vessel plane of the fetal heart, namely, the aorta, the pulmonary artery and the superior vena cava. We began by carefully selecting the most promising baseline model for the segmentation of three-vessel cross-sectional images. Subsequently, we validated the effectiveness of region-of-interest (ROI) detection before segmentation, as many studies have demonstrated that ROI detection followed by segmentation can significantly enhance the segmentation of small objects [34, 35]. Lastly, we devised an attention-based multi-scale feature extraction module to address the challenge posed by the large variation in vessel sizes. In comparison to several existing deep learning methods, our proposed framework demonstrates best performance in segmenting the three-vessel plane of the fetal heart. Our method holds the potential to assist sonographers in enhancing the effectiveness and accuracy of vessel assessment during fetal heart examinations.

## Results

### Comparison of baseline models for full-size 3VV image segmentation

We first conducted a comparative experiment to assess the performance of several baseline segmentation models on full-size fetal 3VV ultrasound images, including FCN [15], U-Net [16], U-Net++ [20], Attention U-Net [21] and Deeplabv3 [22]. Table 1 presents the results of these baseline segmentation models on our collected dataset of fetal heart 3VV images. As shown in Table 1, Deeplabv3 surpasses the other models in segmenting all three vessels, achieving the highest Dice and IoU scores and the lowest HD scores [36]. While Deeplabv3 has exhibited strong performance in PA and Ao segmentation, its performance in segmenting the SVC remains suboptimal, primarily due to the SVC’s small size.

**Table 1:** Comparative analysis of baseline models for three-vessel segmentation in full-size 3VV images

Baseline	Dice			Mean		
	PA	Ao	SVC	IoU	HD	Dice
FCN [15]	78.81	75.84	54.55	58.61	3.95	69.73
U-Net [16]	80.75	85.10	68.40	67.56	3.65	78.08
U-Net++ [20]	82.38	86.60	69.45	69.78	3.56	79.48
Attention U-Net [21]	81.73	84.91	72.80	69.29	3.63	79.82
Deeplabv3 [22]	**83.49**	**86.61**	**73.36**	**70.74**	**3.50**	**81.15**

PA: pulmonary artery; Ao: aorta; SVC: superior vena cava; Mean: the average value of the three vessels

The optimal value is highlighted in bold, while the second-best value is underscored (in column)

### Evaluation of the two-stage framework: ROI detection followed by segmentation

**Fig. 1:**
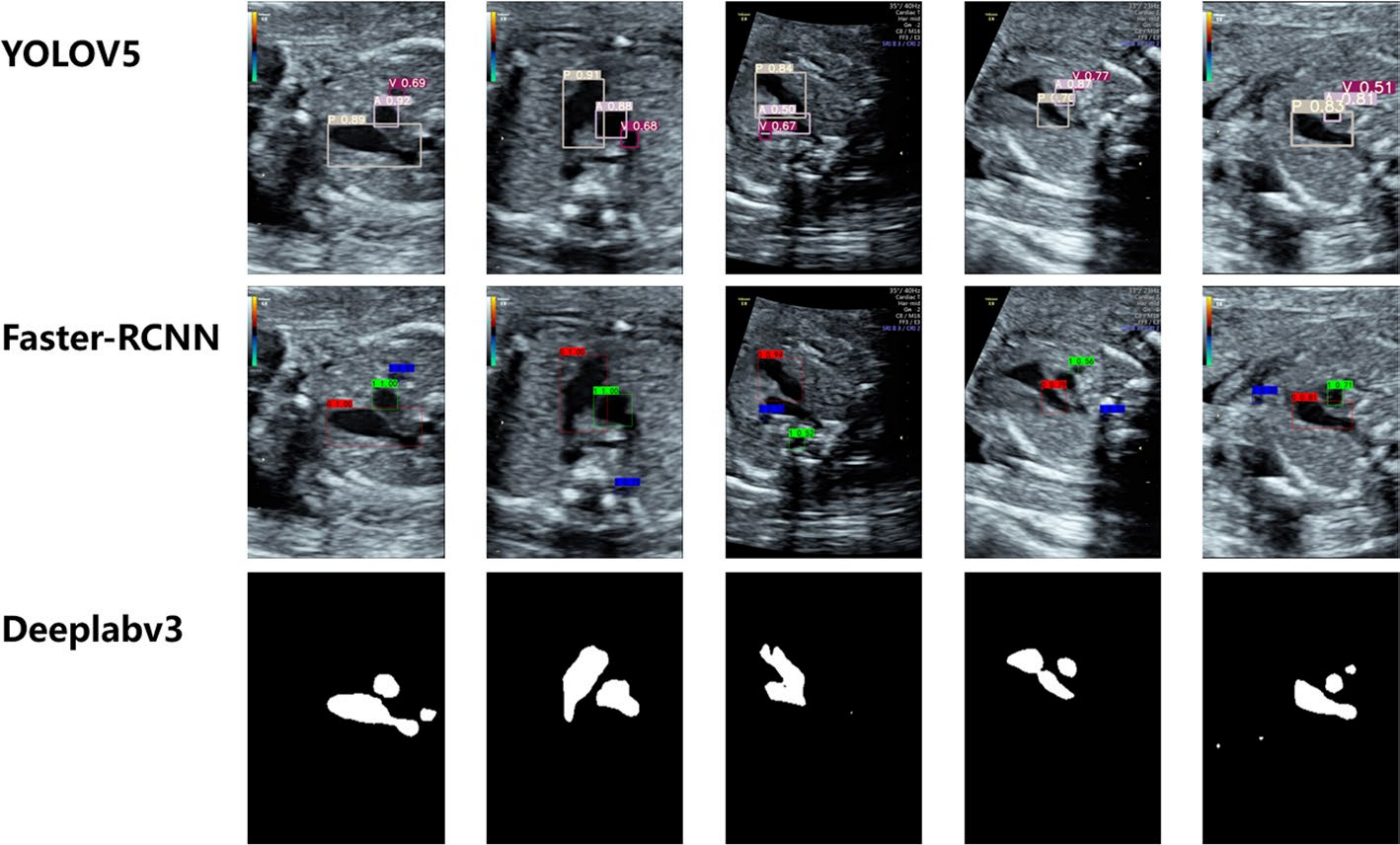
Visual comparison of different ROI localization strategies

In this section, we evaluated the effectiveness of ROI detection in relation to its subsequent segmentation. We compared three ROI localization strategies for our task, and the results are presented in Table 2. “Deeplabv3 + Deeplabv3” represents a two-stage framework comprising two Deeplabv3 models, with the first performing a binary segmentation for ROI localization and the second performing a multi-class segmentation for a fine extraction of the three vessels. “Faster-RCNN + Deeplabv3” is a framework where ROI detection is carried out by Faster-RCNN, followed by subsequent segmentation with Deeplabv3. “Yolov5 + Deeplabv3” is a framework in which ROI detection is performed using Yolov5, followed by subsequent segmentation with Deeplabv3. As shown in Table 2, the two-stage framework “Yolov5 + Deeplabv3” demonstrates superior performance compared to other frameworks for our task. In comparison to the baseline one-stage method, which involves using Deeplabv3 for full-size image segmentation, "Yolov5 + Deeplabv3" enhances the Dice scores of PA, Ao, and SVC by 1.95%, 0.74%, and 3.18%, respectively. Figure 1 provides exemplary results of various ROI detection strategies.

**Table 2:** Comparative analysis of two-stage frameworks for vessel segmentation in fetal 3VV ultrasound images using varied ROI localization strategies

Method	Dice			Mean		
	PA	Ao	SVC	IoU	HD	Dice
Deeplabv3 (full-size)	83.49	86.61	73.36	70.74	3.50	81.15
Deeplabv3 + Deeplabv3	82.71	83.79	66.15	67.92	3.58	77.55
Faster-RCNN + Deeplabv3	72.03	67.36	61.42	57.59	4.14	66.93
Yolov5 + Deeplabv3	**85.44**	**87.35**	**76.52**	**73.25**	**3.30**	**83.11**

The optimal value is highlighted in bold (in column)

### Comparison of our method with state-of-the-art segmentation models

**Fig. 2:**
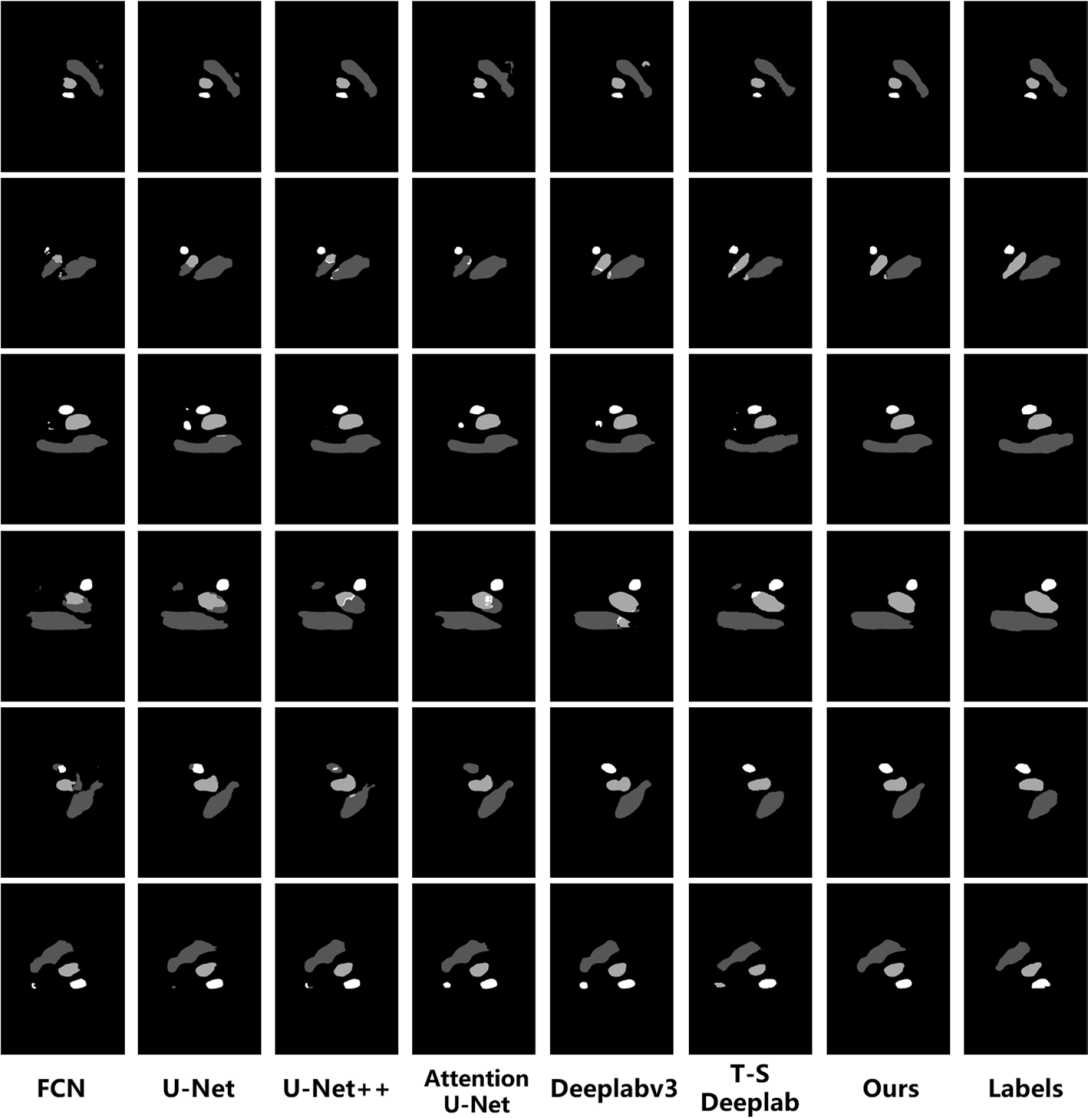
Visual comparison of the performance of different segmentation models in our task

Our experiments have demonstrated that the two-stage framework, where in Yolov5 detection followed by Deeplabv3 segmentation, exhibits superior performance in our task. To further enhance the segmentation performance, we replace the Atrous Spatial Pyramid Pooling (ASPP) module in Deeplabv3 with a novel module called "Attentional Multi-scale Feature Fusion (AMFF)". As shown in Table 3, our proposed method significantly outperforms existing CNN-based segmentation models in the segmentation of all three vessels in fetal heart ultrasound images across all evaluation metrics. Compared to the original Deeplabv3, our model increases the Dice score for Ao by 1.77% and for SVC by 1.02%. Figure 2 provides a visual comparison of the segmentation performance of different methods in our task.

**Table 3:** Comparison of different segmentation models for vessel segmentation in YOLOv5-generated ROIs

Method	Dice			Mean		
	PA	Ao	SVC	IoU	HD	Dice
FCN [15]	82.90	81.64	69.03	67.40	3.54	77.86
U-Net [16]	83.80	83.28	72.94	70.18	3.48	80.01
U-Net++ [20]	83.31	84.25	72.41	69.92	3.47	79.99
Attention U-Net [21]	82.93	81.41	66.72	67.13	3.61	77.02
Deeplabv3 [22]	85.44	87.35	76.52	73.25	3.30	83.11
T-S-deeplab [32]	81.36	86.89	74.24	71.38	3.46	81.35
Ours	**85.55**	**89.12**	**77.54**	**74.51**	**3.25**	**84.07**

The optimal value is highlighted in bold, while the suboptimal value is underlined (in column)

Comparing with the underlined values, this further illustrates the superiority of our proposed method

## Discussion

In this study, we propose a deep-learning based framework for the automatic identification and segmentation of the PA, Ao, and SVC in fetal heart 3VV ultrasound images. We conducted a performance comparison of several baseline segmentation models in our specific task and our method has the potential to assist physicians in diagnosing congenital heart defects more effectively and objectively in clinical practice. Specifically, the automatic segmentation of vessels in the 3VV of the fetal heart could assist in-experienced physicians to efficiently localize the three vessels. In addition, it was a prerequisite for developing a technique for the automatic measurement of the size ratio between the pulmonary artery and the aorta, a biometric essential for screening CHD.

One challenge in our task is the large variation in the size of vessels. Compared with the other two vessels, the SVC is much smaller, thus often being overlooked by a multi-object segmentation model. We conducted a performance comparison of several baseline segmentation models, including FCN, U-Net, U-Net++, Attention U-Net, and Deeplabv3, using our collection of full-sized ultrasound images. As shown in Table 1, Deeplabv3 outperforms the other models thanks to its multiscale-feature extraction mechanism that incorporates varying receptive field sizes, rendering it advantageous for capturing features of vessels with diverse scales.

Another challenge in segmenting the 3VV images is the interference caused by the surrounding background [37]. This is particularly problematic for small-sized SVC vessels, which often occupy a limited region in the image. To mitigate the adverse effects of the irrelevant background information and allow the segmentation model to concentrate on vessel details, we employed a two-stage framework, with the first stage for detecting the ROI, and the second stage for a fine segmentation of the three vessels in the cropped ROI images. We experimented with different ROI extraction strategies in combination with the Deeplabv3 segmentation model. Our findings, shown in Table 2, indicate that the combination of Yolov5 and Deeplabv3 produced the most optimal segmentation performance, while the combination of Faster R-CNN and Deeplabv3 yielded the poorest results. A visual analysis of Fig. 1 revealed that the discrepancy can be attributed to Faster R-CNN's higher tendency for false positives during the detection phase, particularly in the inaccurate recognition of the smallest SVC. This inaccuracy led to a significant error in ROI extraction, subsequently hindering satisfactory segmentation results in the second stage. Notably, the performance of Deeplabv3 combined with Deeplabv3 was even worse than directly performing a multi-class segmentation task on full-sized images. Similarly, based on visual analysis of the results in Fig. 1, Deeplabv3 exhibited a higher degree of difficulty in identifying the SVC during initial ROI extraction for foreground–background separation. The loss of SVC in the ROI extraction stage makes it challenging for Deeplabv3 to achieve effective segmentation in the second stage.

To further improve the segmentation performance, we replaced the ASPP module in the Deeplabv3 it with our designed AMFF (Attentional Multi-scale Feature Fusion) module. Specifically, we devised multi-scale feature extraction branches with varied dilation rates, and flow small-scale features through different branches using hierarchical connections. We also introduced a spatial attention mechanism at the end of each branch to further enhance feature representations. These modifications allowed the model to effectively capture multi-scale features of all blood vessels in our task. As demonstrated in Table 3, within the two-stage framework, the integration of our AMFF module into Deeplabv3 resulted in significant improvements in the segmentation of both the Ao and SVC.

While our framework has exhibited promising outcomes, this study has two limitations. First, the data size is restricted, and all data are obtained from a single hospital, lacking external validation data from other medical facilities. As a result, one of our future objectives involves validating our model on a larger and more diverse dataset. Second, our 3VV segmentation framework is not trained end-to-end. It consists of an ROI extraction model and a segmentation model, each trained independently. In the future, we aim to improve our method by transitioning to a unified framework, thereby enhancing efficiency in both training and application.

## Conclusions

In this study, we propose a two-stage deep learning framework for vessel segmentation in fetal 3VV ultrasound images, involving a Yolov5 for ROI localization and a Deeplabv3 model equipped with our novel AMFF module for segmentation within the ROI regions. Our proposed framework has exhibited remarkable performance in segmenting all three vessels with average HD value of 3.25 and Dice value of 84.07% and IoU value of 74.51%, surpassing other state-of-the-art segmentation models. Our future work includes validating our method on a larger and more diverse dataset collected from multiple hospitals to enhance the generalizability of our approach.

## Methods

**Fig. 3:**
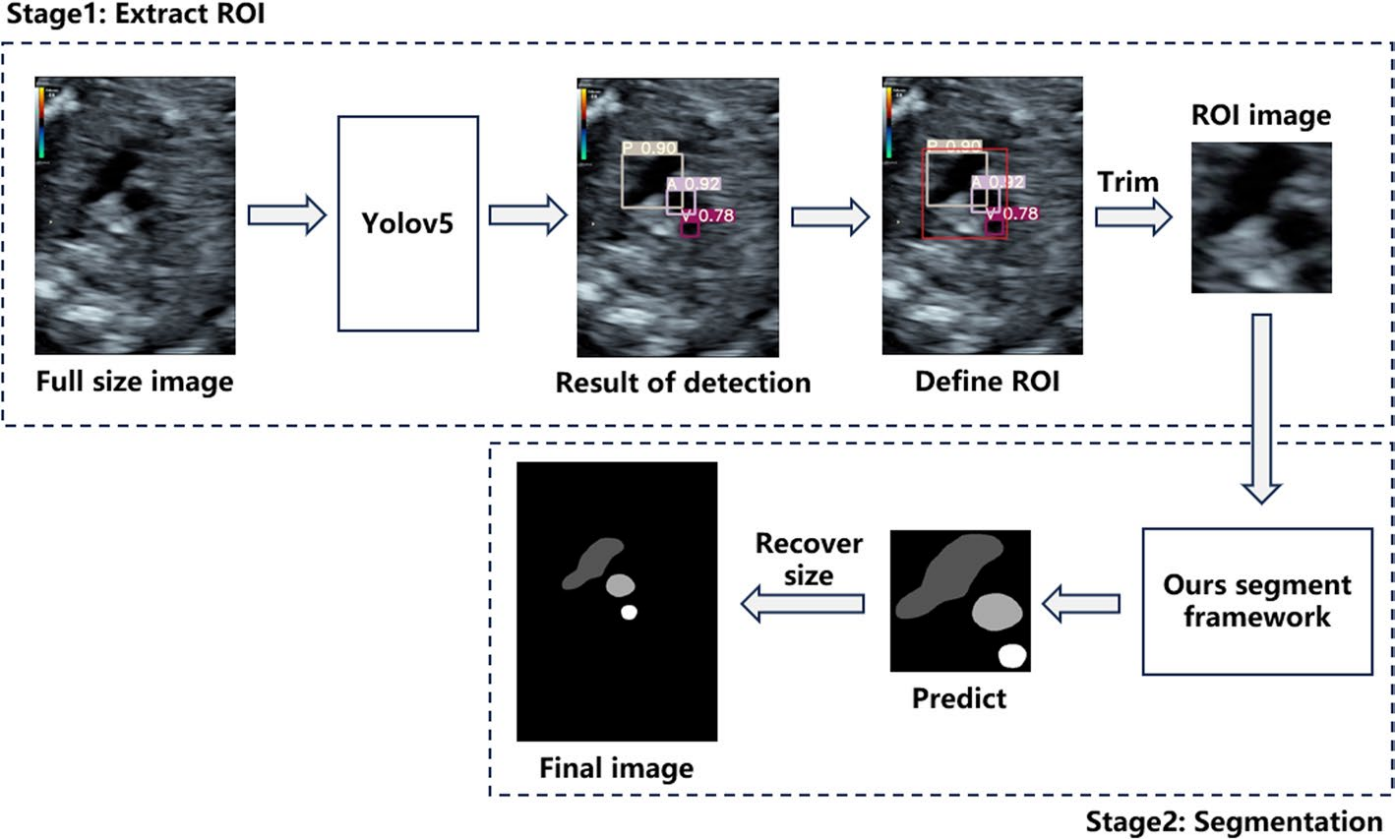
Workflow of our proposed strategy

In this paper, we developed a two-stage deep-learning framework for the identification and segmentation of the vessels in fetal heart 3VV images to assist radiologists in diagnosing vascular structural abnormalities. The overall workflow of our method is illustrated in Fig. 3.

The code and detailed instructions for users have been made available to the public at the following link: https://github.com/ylfas/3VV_demo/blob/master/README.md.

### Clinical dataset

The dataset used in this study was obtained from Hangzhou Normal University Affiliated Xiaoshan Hospital, China, with ethical approval granted by the hospital’s Ethics Committee. The dataset comprises images acquired from 607 pregnant women in mid-term pregnancy, with gestational ages ranging from 20 to 40 weeks. The images were captured using a GE Voluson E8 ultrasound machine equipped with a 2–5 MHz linear ultrasound transducer. During the examination process, physicians conducted a comprehensive assessment of the fetal cardiac structure and function. Standard cardiac planes, including the 3VV, were captured and stored. All patient information in the images was de-identified. After excluding non-standard or challenging-to-interpret sectional images, a refined set of 511 images was obtained, including 413 normal cases and 98 abnormal cases. Table 4 presents the distribution of various types of CHD data reported within our final dataset. Subsequently, these images were labeled by two experienced physicians with 15 and 20 years of expertise, respectively. The two physicians independently annotated the boundaries of the vessels within the 3VV images. If there were notable disparities between their annotations, a consensus was reached through a comprehensive discussion. The final mask label for each image was derived by averaging the annotations of the two physicians.

**Table 4:** Types of CHD reported in our dataset

Data type	Number
Normal images	413
Abnormal images	98
Abnormal vessel diameter ratio	67
Cardiac chamber abnormality	6
Arterial vascular abnormality	15
Outflow tract abnormality	3
Tetralogy of Fallot	7

### Data preprocessing

The dataset was divided into training, validation, and test sets in a ratio of 7:1:2. Before being fed into a network, all images were resized to 256 × 256 and subjected standard normalization. Data augmentation techniques such as random horizontal flipping, random angle rotations, and random scale adjustments are applied to mitigate over-fitting [38].

### ROI localization

Many studies have demonstrated that a two-stage deep learning framework that involves ROI detection followed by segmentation, can significantly enhance the final segmentation performance, particularly for small objects [34, 35]. Specifically, the first stage of this framework aims to localize the target objects in the original images. This localization can be accomplished using an object detection model, such as Faster-RCNN [39], and YoLo-series models [40–42]. It can also be achieved through a segmentation model applied to full-size images to obtain coarse object masks [43, 44]. The areas containing the coarse masks are treated as ROIs. In the second stage, the ROI regions are cropped from the original images and fed into a second model to achieve fine object segmentation. This two-stage approach can mitigate the adverse effects of irrelevant background and enable the model to concentrate on object details, thus improving the segmentation performance.

We compared three different ROI localization strategies on our dataset. The first strategy involves using Deeplabv3 to roughly segment the full-size image. The ROI region was then defined by expanding the segmented vessel masks. The ground truth labels in this method were binary vessel masks, where all three vessels were labeled with 1 and the background was labeled with 0. The second strategy utilized YOLOv5 to identify the three vessels within the full-size image. From the predicted candidate boxes for each class, the one with the highest confidence was selected as the final output. This process yielded three candidate boxes, each containing one of the three vessels. The minimum bounding rectangle that enclosed all three predicted boxes was extended by 5 pixels to obtain the final ROI of the image (as shown in Fig. 3). The third strategy was similar to the second strategy, but instead employed Faster RCNN [39] as the detection model. In the latter two strategies, ground truth labels were bounding boxes of the three vessels.

### Attention-based multiscale feature fusion framework for vessel segmentation

**Fig. 4:**
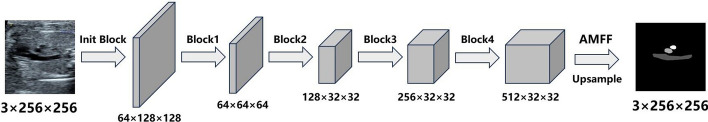
The framework of modified network based on deeplabv3. AMFF: Attentional Multi-scale Feature Fusion module

The second stage of our framework is a modified Deeplabv3 equipped with our novel AMFF module for instance segmentation of the three vessels. The ROI regions are cropped from the original images and fed into the second model to achieve fine object segmentation. For the second model training, the label format comprises boundary masks for each blood vessel within the cropped region of each ultrasound image from the initial stage, with individual differentiation of each blood vessel as a distinct category. The AMMF’s architecture is illustrated in Fig. 4. A cascade of ResNet34 [17] blocks are used to encode image features. To be concrete, the initial phase involves an initialization block, which consists of a 7 × 7 convolution with a stride of 2, a padding of 3, and a Batch Normalization (BN) layer. Following the initialization block, multiple copies of the last ResNet34 block that referred to as blocks 1 to 4 in Fig. 4 are employed and organized in a cascading manner. These blocks contain four 3 × 3 convolutions, with the first convolution having a stride of 2, except for block 3 and block 4. The resulting deep features are subsequently input into our specially designed AMFF module to enhance feature representations across various scales.

**Fig. 5:**
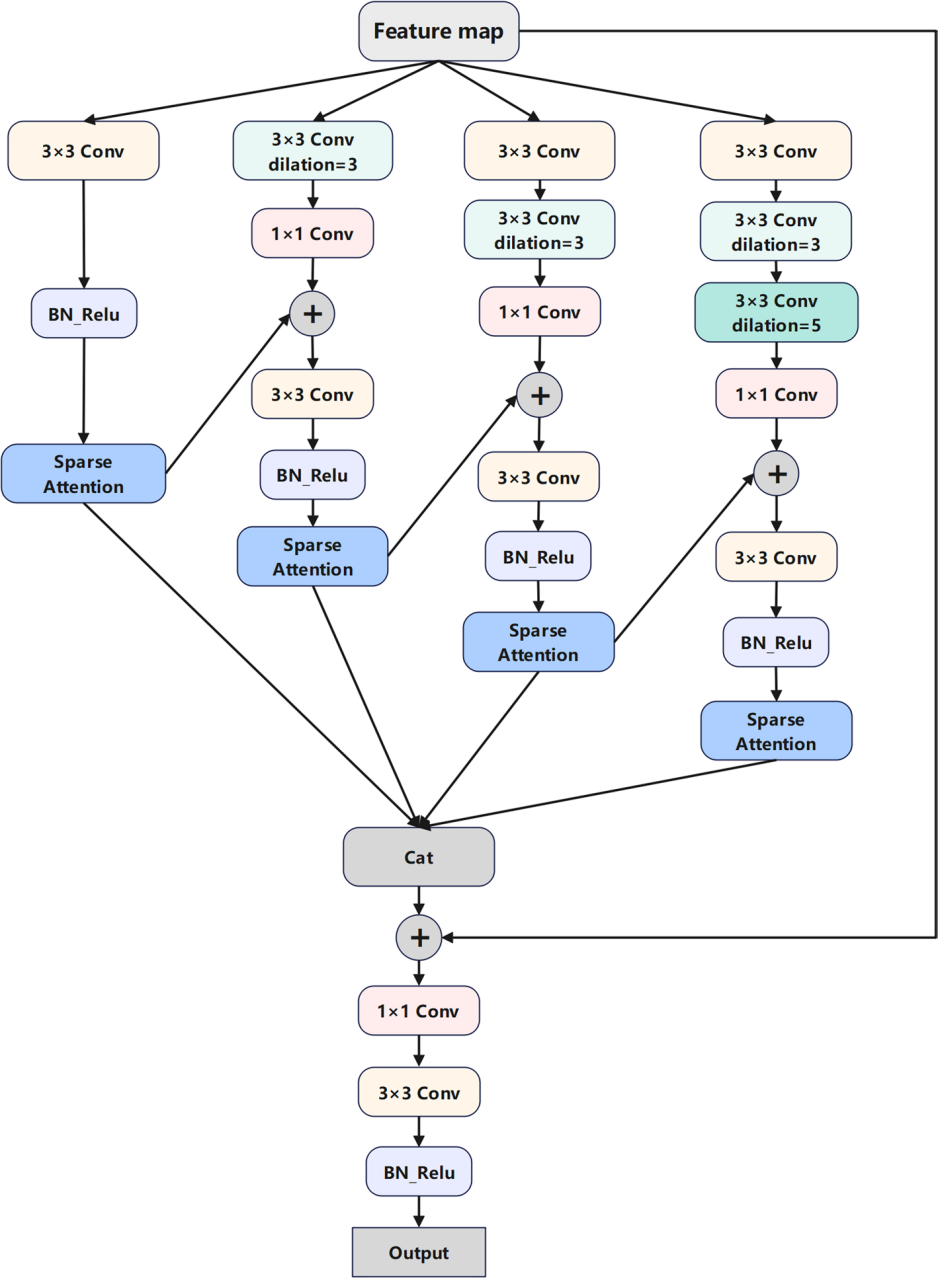
Structure of AMFF (Attentional Multi-scale Feature Fusion) module

Figure 5 displays the structure of our AMFF module. It consists of multiple feature extraction branches with convolutions of different dilation rates to obtain features with diverse receptive fields. To ensure that each branch preserves small object features, we encourage interaction among branches by integrating features through hierarchical connections. Furthermore, we introduce spatial attention operations to selectively enhance the most effective features of each branch, thereby improving feature representations at multiple scales. Subsequently, the features from all branches are concatenated to create fused features that retain information related to multi-scale targets. The fused feature (2048 × 32 × 32) is then dimensionally reduced to three channels through two convolutional layers, with each channel predicting one type of vessel. Finally, the prediction is upsampled eight times through bilinear interpolation to restore it to the original image resolution.

The loss function for training the model is a combination of cross-entropy loss and dice loss, which is defined as:

1 $$L=0.5*{L}_{{\text{dice}}}+0.5*{L}_{ce}$$

2 $${L}_{ce}=-\frac{1}{MN}\sum_{k}^{M}\sum_{i}^{N}{W}_{k}\cdot g\left(k,i\right)\cdot {\text{log}}\left(p\left(k,i\right)\right), {W}_{k}=\frac{{N}_{{\text{total}}}}{{N}_{k}}$$

3 $${L}_{{\text{dice}}}=\frac{1}{MN}\sum_{k}^{M}\left(1-2\frac{\sum_{i}^{N}g\left(k,i\right)\cdot p\left(k,i\right)}{\sum_{i}^{N}g\left(k,i\right)+\sum_{i}^{N}p\left(k,i\right)}\right)$$

In above equations, the symbol *k* represents the index of the *k*-th channel, and the symbol *i* denotes the position of the *i*-th pixel within each channel. Therefore, the symbol *p*(*k*, *i*) is used to denote the prediction of the *i*-th pixel in the *k*-th channel of the matrix, while the symbol *g*(*k*, *i*) is employed to represent the *i*-th pixel in the *k*-th channel of the actual segmentation mask.

### Model comparison and evaluation metrics

To select the best-performing baseline model as the foundation for this study, we first conducted a comparative experiment to assess the performance of several baseline segmentation models on full-size fetal 3VV ultrasound images, including FCN [15], U-Net [16], U-Net++ [20], Attention U-Net [21] and Deeplabv3 [22]. All models underwent training and evaluation using the identical data-splitting strategy (70% training, 10% validation, 20% test) and hyperparameter settings. To be specific, all models were trained for a total of 35 epochs using the Adam optimizer [45]. The learning rate was decreased from the initial value of 0.001to 0.0001 in the final 10 epochs, with a decay rate of 1e−8. The loss function is a combination of cross-entropy loss and Dice loss, with both losses weighted equally at 0.5 [46].

In this study, we employed several metrics to evaluate the segmentation performance of different methods, including IoU, HD, and Dice coefficient. IoU is defined as the ratio of the overlap area between the predicted and ground truth masks to the area of their union. It quantifies the spatial overlap between the two masks, providing insight into segmentation accuracy. HD represents the maximum distance between the predicted and ground truth boundaries. It offers a measure of the maximum segmentation error, helping us understand the extent of boundary discrepancies. The Dice coefficient is calculated as twice the intersection of the predicted and true masks divided by the sum of their areas. It serves as a metric for assessing the agreement between the predicted and true masks and is commonly used in medical segmentation tasks. These metrics collectively offer a comprehensive evaluation of the segmentation performance, aiding in the assessment of the accuracy and effectiveness of our method across different vessels [36]. The three metrics are defined as follows:

4 $${\text{IOU}}=\frac{\mathrm{Intersection\ Area}}{\mathrm{Union\ Area}}=\frac{{\text{TP}}}{{\text{TP}}+{\text{FN}}+{\text{FP}}}$$

5 $${\text{Dice}}=\frac{2*{\text{TP}}}{2*{\text{TP}}+{\text{FN}}+{\text{FP}}}$$

In the Eqs. (4) and (5), TP (True Positives) represents the number of observations correctly predicted as the positive class. TN (True Negatives) represents the number of observations correctly predicted as the negative class. FP (False Positives) indicates the number of observations that were incorrectly predicted as the positive class. FN (False Negatives) represents the number of observations that were incorrectly predicted as the negative class.

6 $${{\text{HD}}}_{A}={{\text{max}}}_{a\in A}{({\text{min}}}_{b\in B}d(a,b))$$

7 $${{\text{HD}}}_{B}={{\text{max}}}_{b\in B}{({\text{min}}}_{a\in A}d(b,a))$$

8 $$\text{HD}(\text{A,B})={\text{max}}({\text{HD}}_{A},{\text{HD}}_{B})$$

In the Eq. (8), A represents the set of points in our predicted matrix, while B represents the set of points in the actual mask label matrix. ${HD}_{A}$ calculates the maximum value of the shortest distances between all points in set A to the points in set B, whereas ${{\text{HD}}}_{B}$ computes the maximum value of the shortest distances between all points in set B to the points in set A. The final Hausdorff distance value, denoted as HD, is determined by selecting the larger of the two calculated values.

## Data Availability

The data sets during the current study are not publicly available due to hospital information protection mechanism, but are available from the corresponding author on reasonable request.

## Abbreviations

CHD: Congenital heart disease
PA: Pulmonary artery
Ao: Aorta
SVC: Superior vena cava
3VV: Three-vessel view
ROI: Region of Interest
AMFF: Attentional Multi-scale Feature Fusion
FCN: Fully Convolutional Network
ASPP: Atrous Spatial Pyramid Pooling
IOU: Intersection over Union
HD: Hausdorff Distance
BN: Batch Normalization
